# The contribution rate of stem–leaf and root of alfalfa (*Medicago sativa L.*) with diverse spatial distribution patterns to sediment and runoff reduction on the Loess Plateau

**DOI:** 10.3389/fpls.2026.1854426

**Published:** 2026-06-01

**Authors:** Chong Yao, Yan Zhuan, Yonghua Liu, Yixin Wang, Gaohan Xu, Siyuan Chen, Ming Zhu, Faqi Wu

**Affiliations:** 1 School of Geographic Sciences, Xinyang Normal University, Xinyang, Henan, China; 2 College of Tea and Food Science, Xinyang Normal University, Xinyang, Henan, China; 3 School of Teacher Education, Xinyang Normal University, Xinyang, Henan, China; 4 Collage of Soil and Water Conservation Science and Engineering, Northwest A&F University, Xianyang, Shaanxi, China

**Keywords:** alfalfa, rainfall simulation, soil erosion, spatial distribution pattern, vegetation component

## Abstract

**Introduction:**

Herbaceous plants regulate the generation of runoff and sediment and the anti-erosion ability of soil by altering vegetation coverage and root systems, thereby influencing soil erosion. However, the variation in soil erosion reduction benefits and the contributions of stem–leaf and roots under diverse vegetation spatial distribution patterns on the Loess Plateau have not yet been understood.

**Methods:**

In the current study, alfalfa spatial distribution patterns with vegetation coverage of 50% (T1 treatment; LS, downstream of hillslopes; MS, middle of hillslopes; US, upper of hillslopes; and SS, equidistant planting), four alfalfa spatial distribution patterns without above-ground biomass (T2 treatment), and bare soil (CK) were designed. Rainfall simulations with slope gradients of 8.75%, 17.63%, 26.80%, and 36.40% and a rainfall intensity of 90 mm h^−1^ were carried out on runoff plots (2 m × 1.0m) filled with clay loam. The initial runoff generation time (RT) was recorded, and runoff sediment samples were collected.

**Results:**

The results showed that the RT for the T1 and T2 treatments was delayed by 1.47 to 2.51 and 0.75 to 1.65 min, respectively, compared with CK. The mean runoff rate for the T1 and T2 treatments ranged from 0.18 to 1.15 and from 0.61 to 1.16 L m^−2^ min^−1^, respectively. The mean sediment rate for the T1 and T2 treatments ranged from 1.50 to 18.63 and 3.01 to 20.01 g m^−2^ min^−1^, respectively. The runoff reduction benefits (RRB) for the T1 and T2 treatments ranged from 44.20% to 84.67% and from 34.40% to 43.66%, respectively. The sediment reduction benefits (SRB) for the T1 and T2 treatments ranged from 25.01% to 86.48% and from 9.30% to 71.30%, respectively. The contribution rates of stem–leaf and root to RRB ranged from 3.87% to 30.14% and from 10.10% to 30.80%, respectively, and those for SRB ranged from 9.75% to 24.65% and from 7.28% to 37.75%, respectively. Principal component analysis indicated that the slope gradient explained the greatest variation in runoff, followed by the spatial distribution pattern and vegetation traits.

**Conclusion:**

The research results provide references for vegetation protection and management in areas with severe soil erosion and enrich the understanding of soil erosion mechanisms.

## 1 Introduction

Vegetation coverage has sharply declined due to multiple disturbances, including excessive human grazing, fire, and animal browsing, which, in turn, had triggered severe soil erosion and ecological crises (De Baets et al., 2007; Parhizkar et al., 2025). Lower vegetation coverage, destruction of above-ground biomass, and soil erosion are the main ecological environmental issues in arid and semi-arid regions, especially in the Loess Plateau of China (Wang et al., 2025b; Yao et al., 2026; Zhou et al., 2025). Through the implementation of vegetation restoration, the regional vegetation coverage has been increased (Feng and Jin, 2024; Huang et al., 2023; Wang et al., 2024a, 2025). As a result of the unique climate and soil conditions in the Loess Plateau, the soil has a weak water storage capacity and a lower soil moisture content, and the limited water resources are insufficient for a full vegetation coverage to be achieved across the entire region (Feng et al., 2016; Tuo et al., 2025; Zhang et al., 2019b). The vegetation spatial distribution pattern and components are crucial in influencing soil erosion (Feng et al., 2018; Hu et al., 2023; Wang et al., 2023). Thus, exploring the influence of vegetation spatial distribution patterns and vegetation components on soil erosion is of great significance for clarifying the influencing mechanism and anti-erosion effects of vegetation.

Herbaceous plants regulate the hydrological processes of hillslopes through multi-layered protection provided by roots, stems, and leaves, further influencing the soil erosion process (Li et al., 2024a; Van Dijk and Bruijnzeel, 2001; Wang et al., 2024d). During rainfall, the exposed soil surface is directly impacted by splashed raindrops, which can be intercepted and buffered by plant stems–leaves, thereby reducing damage to the soil structure (Fu et al., 2020; Peng et al., 2026; Wang et al., 2024d). As a result of the shielding effect of leaves, the falling speed and kinetic energy of raindrops are significantly reduced, effectively weakening the fragmentation of soil aggregates (Di Prima et al., 2026; Ma et al., 2015; Zhu et al., 2024a). Plant roots and stems increase soil surface random roughness and slow down the runoff velocity, reducing runoff kinetic energy and concentrated flow erosion (Liu et al., 2022; Wang et al., 2022b). Meanwhile, the water storage capacity of plant litter can increase soil infiltration and reduce hillslope runoff volume (Niu et al., 2024; Wang et al., 2023). However, the contribution of vegetation components to soil erosion reduction varies with the vegetation spatial distribution pattern, and this still requires further verification.

Root development alters soil properties through chemical bonding and physical binding (Gyssels et al., 2005; Wang and Zhang, 2017; Wang et al., 2026). Plant roots tightly bind soil particles through physical interpenetration and entanglement, forming root–soil complex and enhancing soil stability (Shuai et al., 2025; Wang et al., 2022a). The formation of root–soil complex in the form of a network structure enhances the soil erosion resistance by more than 20% (Saadati et al., 2023; Wang et al., 2025c, 2024). The root system squeezes the soil, forming soil micro-channels and enhancing soil permeability (Leung et al., 2017; Niu et al., 2024). Root exudates promote the formation of soil aggregation and organic matter content, significantly regulating soil properties and enhancing soil anti-erosion ability (Li et al., 2017; Nardi et al., 2002). While the regulatory effects of root systems on soil properties and soil erosion have been quantified, further research is needed to investigate soil erosion variation with vegetation spatial distribution pattern.

Vegetation spatial distribution pattern refers to the spatial configuration of vegetation with varying quantities, sizes, and types within a region, which alters soil erosion by changing the runoff path and runoff velocity and intercepting sediment (Feng et al., 2018; Puigdefábregas, 2005; Wang et al., 2014). The water–sediment relationship, hydrological connectivity, and sediment transport capacity on hillslopes are closely related to vegetation patterns. A well-configured vegetation pattern slows down the runoff velocity by using stems for blocking and leaves for dragging and prolongs the infiltration time to reduce the runoff volume (Feng and Jin, 2024; Puigdefábregas, 2005; Zhang et al., 2019a). Vegetation community spatial heterogeneity can change the runoff path, avoiding the formation of concentrated flow and reducing the flow shear stress of overland flow (Hu et al., 2023; Wang et al., 2023). The regulating effect of vegetation patches on soil erosion varies with the position of vegetation patches on hillslopes and the coarseness of the vegetation pattern. Generally, vegetation patches on hillslopes could play a buffering role, thereby effectively reducing runoff and sediment. Vegetation located at the bottom of hillslopes exhibits better soil erosion reduction effects than vegetation located at the top of hillslopes (Feng et al., 2018; Puigdefábregas, 2005). An increase in vegetation pattern coarseness is associated with an increase in hillslope runoff and sediment yield, while greater vegetation patch density leads to less runoff and a stronger ability to retain soil particles.

Since the implementation of Grain for Green and vegetation restoration projects in China in recent years, the ecological environment has been improved and soil erosion has been greatly alleviated (Huang et al., 2023; Yang et al., 2025; Yibin et al., 2024). Alfalfa has a well-developed vertical root system and is highly drought-resistant and tolerant of poor soil conditions (Yao et al., 2023). It not only meets the needs of regional ecological restoration but also provides high-quality forage support for local livestock farming (Xiao et al., 2015). However, the regulatory mechanism of alfalfa with diverse spatial distribution patterns on soil erosion is not yet clear, and the contribution rates of stem–leaf and roots in alfalfa with diverse spatial distribution patterns to soil erosion reduction benefits are still lacking.

Thus, four different alfalfa spatial distribution patterns (LS, downstream of hillslopes; MS, middle of hillslopes; US, upper of hillslopes; and SS, equidistant planting) and bare soil (CK) were designed, and the above-ground biomass was cleared in the current study. Rainfall simulation tests were conducted on runoff plots with different treatments. The purpose of this study is to (1) explore differences in soil erosion processes under diverse alfalfa spatial distribution pattern, (2) quantify the runoff and sediment reduction benefits of alfalfa, and (3) clarify the contribution rate of vegetation components to runoff and sediment reduction benefits under different alfalfa spatial distribution patterns.

## 2 Materials and methods

### 2.1 Study area

Yangling (34°14′–34°20′ N, 107°59′–108°08′ E) is located in the central part of Shaanxi Province, the southern part of the Loess Plateau. It has an altitude of 468 m and a semi-humid continental monsoon climate, with an annual average temperature of 12.9°C and rainfall of 635 to 646 mm. Furthermore, 60% to 70% of rainfall occurs during the rainy season, mainly in the form of short-term heavy rains (Yao et al., 2025; Zhu et al., 2026). The dominant land use type in the study area is mainly agricultural land.

### 2.2 Experimental apparatus

**Figure 1:**
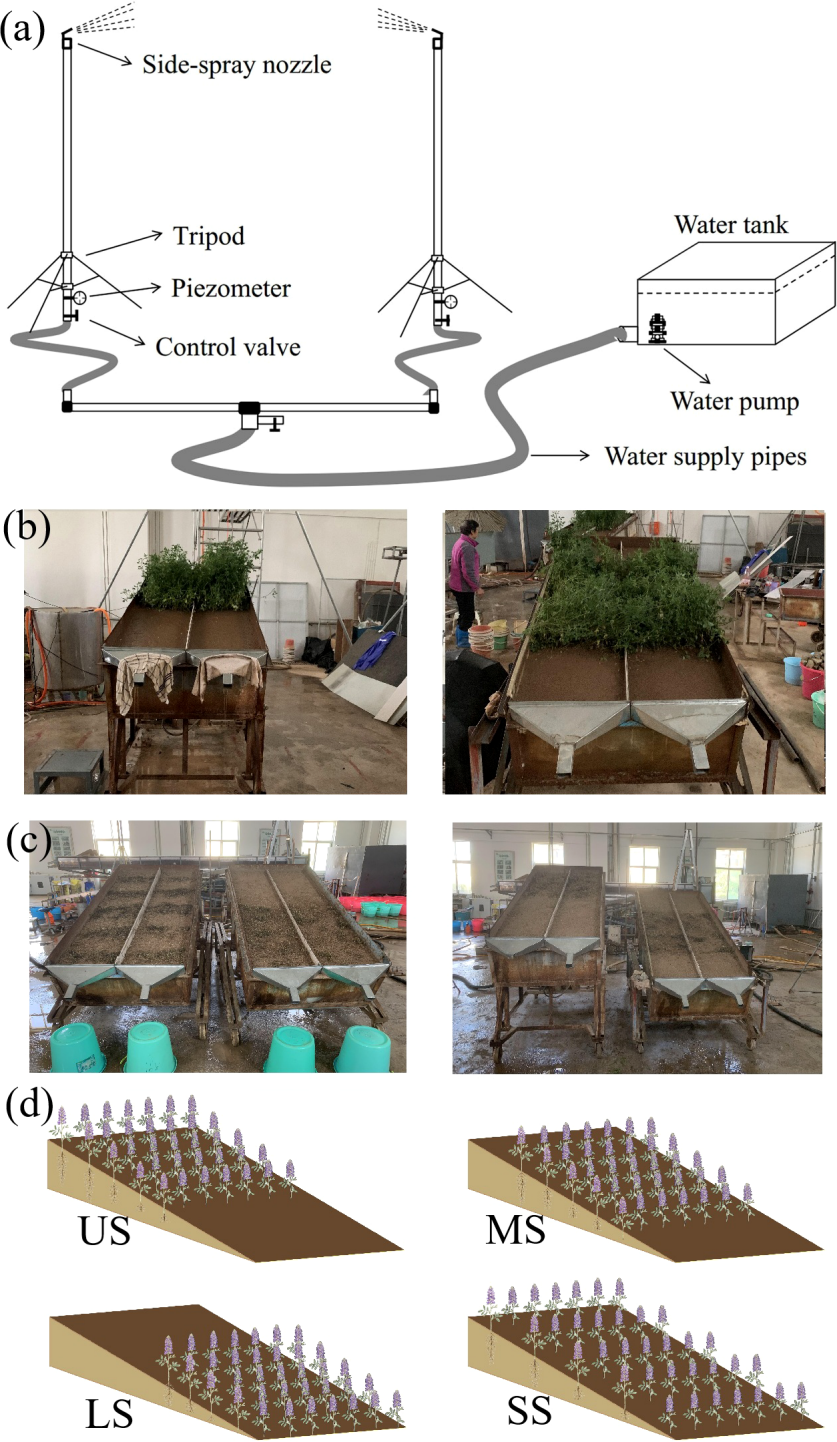
Schematic presentation of the side-spray rainfall simulation system **(a)**, T1 treatment **(b)**, T2 treatment **(c)**, and schematic diagrams of alfalfa spatial distribution patterns **(d)**: US (upper of hillslopes), MS (middle of hillslopes), LS (lower of hillslopes), and SS (equidistant planting).

The rainfall simulation equipment in the current study was a side-spray rainfall machine (**Figure 1**). The entire rainfall machine consists of a water supply system, two independent rainfall support frames, a water supply pressure control device, and a side-spray nozzle. The water supply system is composed of a water tank, a water pump, and a supply pipeline. The height of this rainfall simulation machine is 7.5 m, with an effective rainfall area of 7 m × 5 m. The rainfall uniformity is over 80%, and the adjustable range of rainfall intensity is 30 to 140 mm h^−1^. The rainfall intensity can be adjusted by regulating the water pressure control valve and replacing the spray nozzle. Before rainfall, the rainfall intensity was calibrated.

### 2.3 Experimental design and treatment

In this study, a soil box with length of 2 m, width of 1 m, and depth of 0.4 m was used for the rainfall simulation. The test soil used in the current study was taken from the topsoil layer (0–20 cm) in a local farmland. After the collected soil was air-dried, it was passed through a 10-mm sieve. During soil box filling, a 5-cm layer of thick sand was firstly laid at the bottom of the soil box to facilitate free drainage. A piece of cotton cloth was laid on the upper part of the sand to isolate the soil. Soil was then filled in a 5-cm layer to maintain a soil bulk density of 1.25 g cm^-3^. After each layer was filled, a wooden board was used to gently level it to eliminate discontinuities between soil layers and maintain soil homogeneity. The prepared soil box was left to stand for 15 days to maintain its natural state. The test grass species was alfalfa, a grass commonly used for hillslope protection. At the end of March, alfalfa seeds were sown in the soil box using the strip sowing method. During the process of sowing the grass seeds, the runoff plots were divided into several 20 × 20-cm sub-regions to ensure the formation of different alfalfa planting patterns. To investigate the differences among vegetation spatial patterns in preventing slope erosion, four different vegetation patterns (T1 treatment) were set up: LS, downstream of hillslopes; MS, middle of hillslopes; US, upper of hillslopes; and SS, equidistant planting. The grass cover area of the different vegetation patterns was controlled to be 50% of the entire hillslopes. To explore the influence of alfalfa roots on soil erosion, the above-ground biomass was removed during the flowering period of alfalfa in the four different vegetation patterns, and this was recorded as T2 treatment. During the process of removing the above-ground biomass, efforts should be made to minimize damage to the surface soil and remove all residual leaf stubble. Meanwhile, bare soil (CK) was set as the control. Considering the growth status, vegetation coverage, and the root system development during the different growth stages of alfalfa, the rainfall simulation experiment was conducted during the flowering period of alfalfa. Based on the topographic conditions and the long-term observation of natural rainfall events in the study area, four slope gradients of 8.75%, 17.63%, 26.80%, and 36.40% and a rainfall intensity of 90 mm h^-1^ were designed for the current study, given that this rainfall intensity had been previously used in experimental research and was found to be representative of natural events based on long-term monitoring (Yao et al., 2023). The duration of the rainfall simulation was 60 min.

### 2.4 Measurement of soil erosion

During the rainfall simulation, the initial runoff generation time (RT, min) was observed and recorded. After runoff generation, plastic buckets were used to collect runoff and sediment samples at 3-min intervals. After the rainfall simulation was completed, the total weight of the collected sample was determined and recorded as *Ma* (g). After sedimentation, the supernatant was discarded, and the collected samples were dried and recorded as *Mb* (g). The runoff rate (RR), sediment rate (SR), and infiltration rate (IR) are calculated using (Equations 1–3):

(1) $$SR=\frac{Mb}{At}$$

(2) $$RR=\frac{Ma-Mb}{1000At}$$

(3) $$IR=\frac{I-h}{At}$$

where *A*, *t*, *I*, and *h* are the area of the runoff plot (m^2^), sampling time (min), rainfall intensity (mm min^−1^), and runoff depth (mm), respectively.

The runoff reduction benefits (RRB) for alfalfa, root, and stem–leaf were recorded as RRA, RRR, and RRS, and the sediment reduction benefits (SRB) for alfalfa, root, and stem–leaf were recorded as SRA, SRR, and SRS and were calculated using Equations 4–9:

(4) $$RRS=\frac{R_{T2}-R_{T1}}{R_{T2}}\times 100\%$$

(5) $$RRR=\frac{R_{T0}-R_{T2}}{R_{T0}}\times 100\%$$

(6) $$RRA=\frac{R_{T0}-R_{T1}}{R_{T0}}\times 100\%$$

(7) $$SRS=\frac{S_{T2}-S_{T1}}{S_{T2}}\times 100\%$$

(8) $$SRR=\frac{S_{T0}-S_{T2}}{S_{T0}}\times 100\%$$

(9) $$SRA=\frac{S_{T0}-S_{T1}}{S_{T0}}\times 100\%$$

where *R_T1_*, *R_T2_*, and *R_T0_* are runoff volume for the T1, T2, and CK treatments and *S_T1_*, *S_T2_*, and *S_T0_* are sediment yield for the T1, T2, and CK treatments. The contribution rates of each factor (stem–leaf, root) for runoff reduction (RCR_i_) and sediment reduction (SCR_i_) were then obtained via Equations 10, 11.

(10) $${\text{RCR}}_{i}=\frac{{RR}_{i}}{\sum {RR}_{i}}\times RRA$$

(11) $${SCR}_{i}=\frac{{SR}_{i}}{\sum {SR}_{i}}\times SRA$$

where *RR_i_* and *SR_i_* are the *RRB* and *SRB* of factor *i*, respectively.

### 2.5 Statistical analysis

The individual effects of slope gradient, vegetation spatial distribution pattern, vegetation coverage, and root on the soil erosion process were evaluated by variance partitioning analysis (VPA), which was conducted using R 4.2.1 (R Core Team, 2021). All figures were created using Origin 2021 software (OriginLab Corporation, 2021).

## 3 Results

### 3.1 Initial runoff generation time

**Figure 2:**
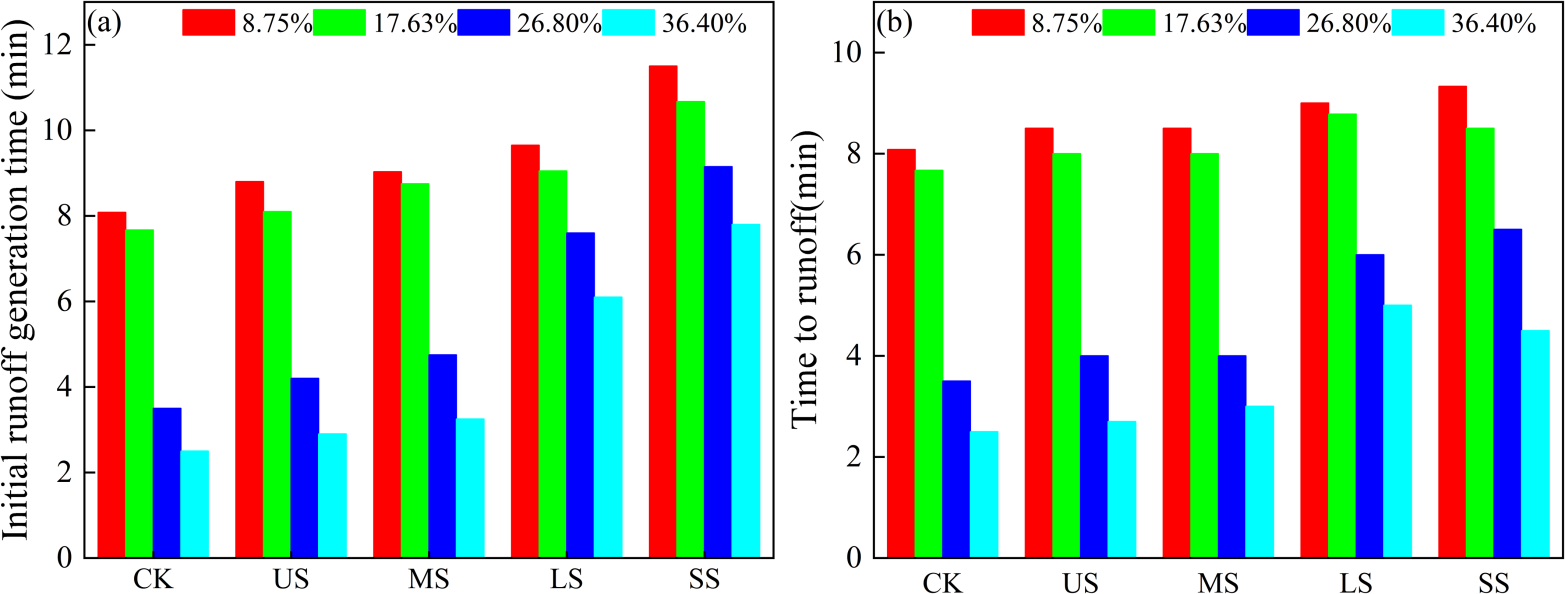
Variations in **(a)** initial runoff generation time and **(b)** time to runoff (min).

The variations in RT on alfalfa-covered hillslopes under the CK, T1, and, T2 treatments are presented in **Figure 2**. At a slope gradient of 8.75%, the RT with the T1 treatment for SS, LS, MS, and US were 8.80, 9.03, 9.65, and 11.50 min, respectively, and those with the T2 treatment for SS, LS, MS, and US were 8.50, 8.50, 9.00, and 9.33 min, respectively. Compared with CK, RT with the T1 and T2 treatments at a slope gradient of 8.75% were delayed by 0.72 to 3.42 min and 0.42 to 1.25 min, respectively. RT with a slope gradient of 36.40% for the T1 treatment for SS, LS, MS, and US were 2.90, 3.25, 6.10, and 7.80 min, respectively, and those with the T2 treatment for SS, LS, MS, and US were 2.7, 3.0, 5.0, and 4.5 min, respectively. RT with the T1 and T2 treatments at a slope gradient of 36.40% were 0.4 to 5.3 min and 0.2 to 2.5 min later than CK, respectively. At a slope gradient of 8.75%, the RT for SS, LS, MS, and US with the T1 treatment was 0.3, 0.53, 0.65, and 2.17 min later than those with the T2 treatment, respectively. RT with a slope gradient of 36.40% for SS, LS, MS, and US with the T1 treatment was 0.2, 0.25, 1.1, and 3.3 min later than those with the T2 treatment, respectively. The results indicated that the RT of alfalfa-covered hillslopes was jointly affected by slope gradient, vegetation spatial distribution pattern, and above-ground biomass.

### 3.2 Soil erosion process

#### 3.2.1 Runoff rate

**Figure 3:**
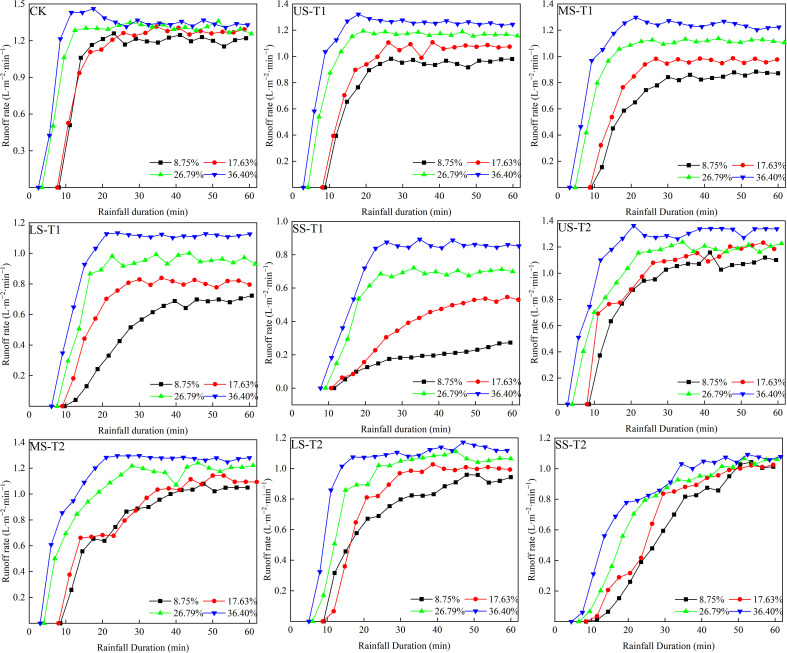
Variations in runoff rate with rainfall duration for different treatments.

The RR of alfalfa-covered hillslopes varies with rainfall duration under different treatments (**Figure 3**). Within the first 10 to 20 min after the onset of rainfall, the RR with different treatments rose rapidly, and then the runoff rates with different treatments reached their peaks. In contrast to CK, the peak runoff rate with the T1 and T2 treatments was lower. Except for the SS pattern with a slope gradient of 8.75% and 17.63%, the runoff rates with different spatial distribution patterns eventually stabilized. The average RR with a slope gradient of 8.75% for the CK and T1 treatment with alfalfa spatial distribution patterns of US, LS, MS, and SS were 1.09, 0.84, 071, 0.48, and 0.18 L m^−2^ min^−1^, respectively, and those for the T2 treatment with US, LS, MS, and SS patterns were 0.91, 0.82, 0.73, and 0.61 L m^−2^ min^−1^, respectively. In contrast to CK, the RR with a slope gradient of 8.75% for the T1 treatment decreased by 22.91% to 83.77% and that for the T2 treatment decreased by 16.58% to 43.67%. The average runoff rates with a slope gradient of 36.40% for the CK and T1 treatment with alfalfa spatial distribution patterns of US, LS, MS, and SS were 1.24, 1.15, 1.11, 0.97, and 0.72 L m^−2^ min^−1^, respectively, and those for the T2 treatment with alfalfa spatial distribution patterns of US, LS, MS, and SS were 1.16, 1.13, 0.99, and 0.82 L m^−2^ min^−1^, respectively. In contrast to CK, the RR with a slope gradient of 36.40% for the T1 treatment decreased by 7.18% to 41.82% and that for the T2 treatment decreased by 6.16% to 34.01%.

#### 3.2.2 Sediment rate

**Figure 4:**
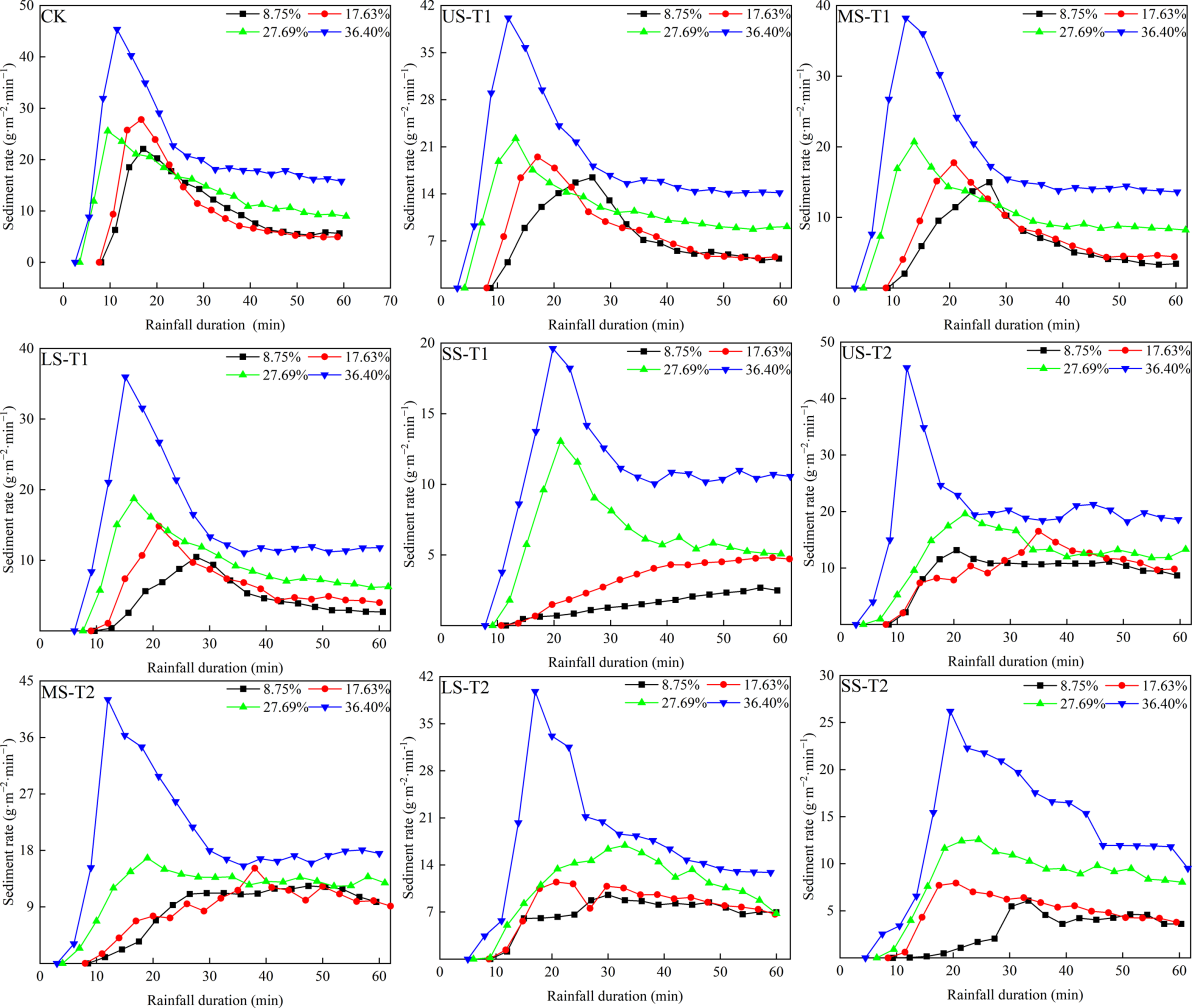
Variations in sediment rate with rainfall duration for different treatments.

The variation in SR with different treatments is shown in **Figure 4**. For the T1 treatment, the SR gradually increased and then tended to stabilize for the LS and SS with a slope gradient of 8.75% and 17.63%, respectively. For other treatments, the sediment rate reached a certain peak and then slowly decreased. The peak sediment rate of CK and alfalfa-covered hillslopes was different—for example, the peak sediment rate for CK with a slope gradient of 8.75% was approximately 20.23 g m^−2^ min^−1^, and that reached 45.35 g m^−2^ min^−1^ at a slope gradient of 36.40%. Moreover, the peak sediment rate appeared earlier with an increase of slope gradient. For the T1 treatment with LS, the peak sediment rate with a slope gradient of 8.75% appeared at approximately 27.65 min, and that with a slope gradient of 36.40% appeared earlier at 15.10 min. At a slope gradient of 8.75%, the average sediment rates of the CK and T1 treatment with the alfalfa spatial distribution patterns of US, LS, MS, and SS were 10.48, 7.86, 6.52, 4.78, and 1.50 g m^−2^ min^−1^, respectively, and those of the T2 treatment with the alfalfa spatial distribution patterns of US, LS, MS, and SS were 9.50, 8.83, 6.83, and 3.01 g m^−2^ min^−1^, respectively. In contrast to CK, the T1 treatment with a slope gradient of 8.75% decreased by 25.01% to 85.69%, and the T2 treatment with alfalfa spatial distribution pattern decreased by 9.30% to 71.30%. At a slope gradient of 36.40%, the mean sediment rates of the CK and T1 treatment with the alfalfa spatial distribution patterns of US, LS, MS, and SS were 21.32, 18.63, 17.88, 10.50, and 10.93 g m^−2^ min^−1^, respectively, and those of the T2 treatment with the alfalfa spatial distribution patterns of US, LS, MS, and SS were 20.01, 19.77, 17.24, and 13.69 g m^−2^ min^−1^, respectively. In contrast to CK, the mean sediment rate with a slope gradient of 36.40% for the T1 treatment decreased by 12.62% to 48.75%, and that for the T2 treatment decreased by 6.15% to 35.80%. The sediment rate and the peak sediment rate varied with the alfalfa spatial distribution patterns and slope gradient.

#### 3.2.3 Infiltration rate

**Figure 5:**
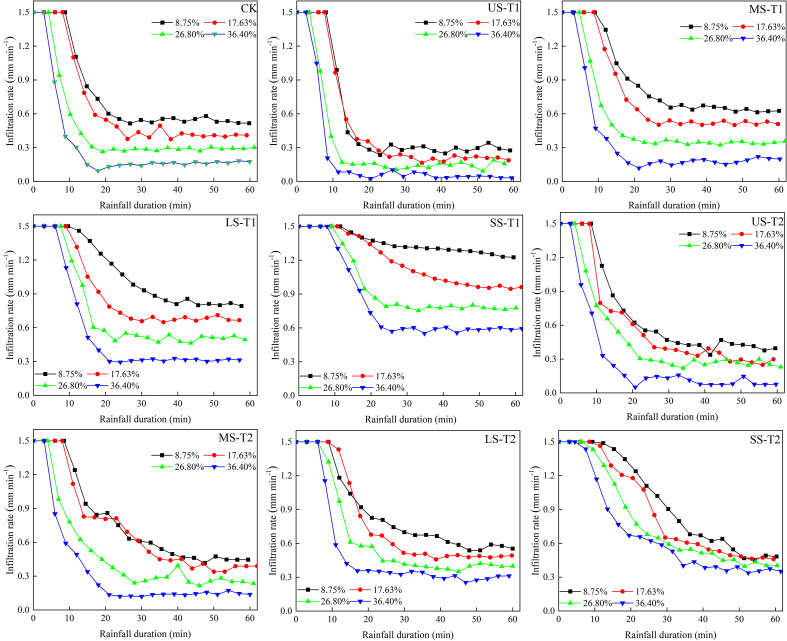
Variations in infiltration rate with rainfall duration for different treatments.

The variation in IR with different treatments is presented in **Figure 5**. The infiltration rate maintained a higher level at onset of rainfall and then decreased. The mean infiltration rates with a slope gradient of 8.75% for the CK and T1 treatment with alfalfa spatial distribution patterns of US, MS, LS, and SS were 0.56, 0.78, 0.89, 1.07, and 1.36 mm m^−2^ min^−1^, respectively, and those for the T2 treatment with alfalfa spatial distribution patterns of US, MS, LS, and SS were 0.72, 0.79, 0.87, and 0.97 mm m^−2^ min^−1^, respectively. In contrast to CK, the mean infiltration rate with slope gradients of 8.75%, 17.63%, 26.80%, and 36.40% for the T1 treatment increased by 38.34% to 141.68%, 31.39% to 125.50%, 31.97% to 162.42%, and 36.55% to 237.82%, respectively, and those for T2 treatment increased by 27.75% to 73.06%, 21.39% to 72.71%, 39.75% to 107.51%, and 31.34% to 188.33%, respectively.

### 3.3 Runoff and sediment reduction benefits

**Figure 6:**
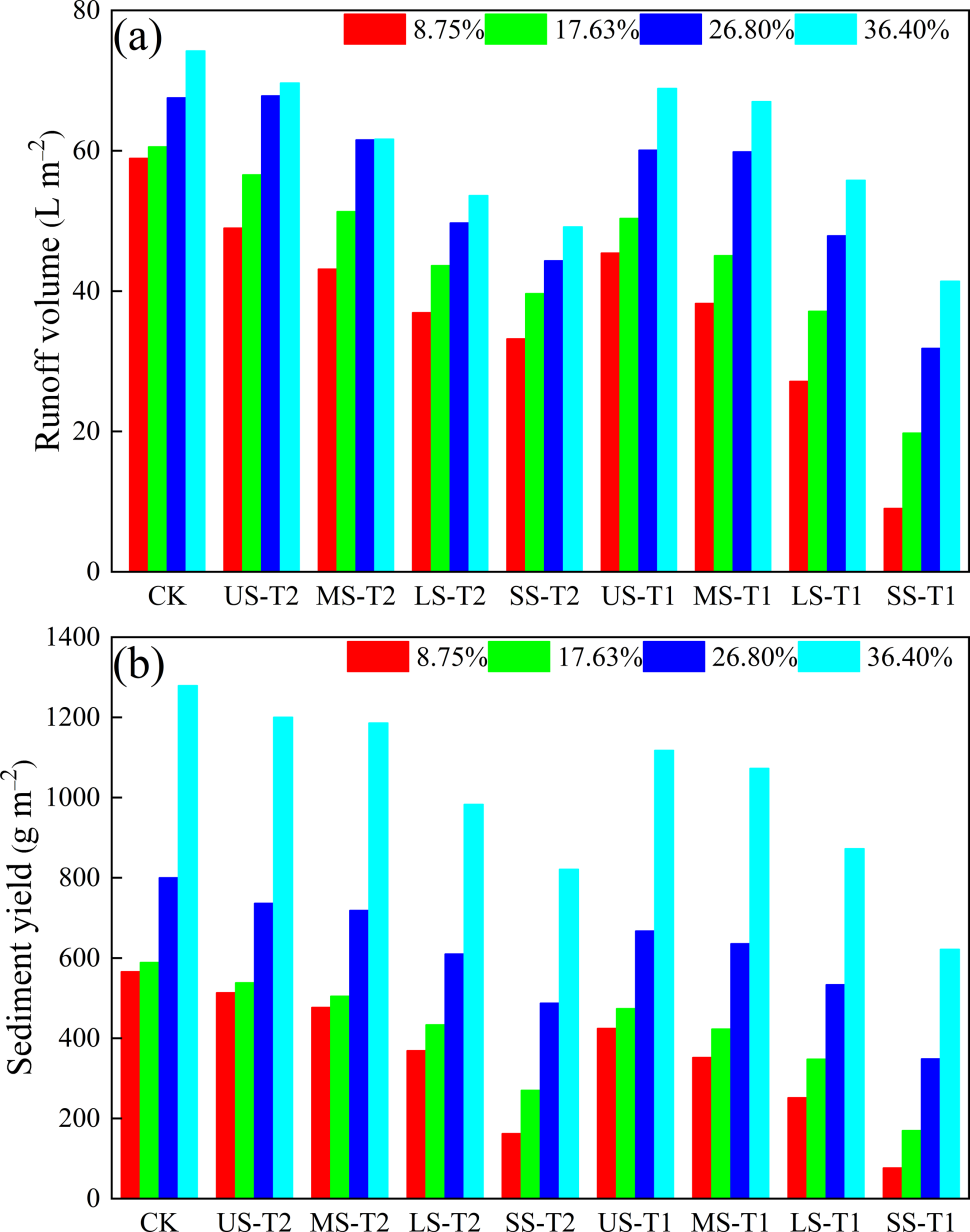
**(a)** Runoff volume (L m⁻²) and **(b)** sediment yield (g m⁻²) under different treatments.

The total infiltration, total runoff volume, and sediment yield for all treatments are presented in **Figure 6**. The total infiltration followed an increasing order of CK, T2, and T1. The total runoff volume and sediment yield for CK ranged from 58.92 to 74.22 L m^−2^ and 565.88 to 1,279.14 g m^−2^, respectively. The total runoff volume and sediment yield followed a decreasing order in CK, T2, and T1, respectively. The runoff and sediment reduction benefits were calculated (**Tables 1**, **2**). In contrast to CK, the total runoff volume for SS under the T1 treatment decreased by 44.20% to 84.67%, and that for the T2 treatment decreased by 34.01% to 43.66%. The variation trend in sediment yield was consistent with runoff volume. Compared with bare soil, the sediment yield for SS under the T1 treatment decreased by 51.40% to 86.48%, and that for T2 treatment decreased by 35.80% to 71.30%.

**Table 1:** The alfalfa, stem- leaf, and root for reduction benefits in runoff (%).

Vegetation pattern	Alfalfa grassland				Root				Stem-leaf			
	8.75%	17.63%	26.80%	36.40%	8.75%	17.63%	26.80%	36.40%	8.75%	17.63%	26.80%	36.40%
SS	84.67	67.35	52.86	44.20	43.66	39.02	36.14	34.01	72.79	46.46	26.18	15.44
LS	53.95	38.71	29.12	24.83	32.70	27.96	24.01	23.79	31.58	14.92	6.72	1.36
MS	35.10	25.61	11.39	9.73	24.77	17.93	8.88	8.62	13.72	9.36	2.76	1.22
US	22.91	16.85	11.06	7.19	16.58	11.48	8.72	6.16	7.59	6.07	2.56	1.09

**Table 2:** The alfalfa, stem- leaf, and root for reduction benefits in sediment (%).

Vegetation pattern	Alfalfa grassland				Root				Stem-leaf			
	8.75%	17.63%	26.80%	36.40%	8.75%	17.63%	26.80%	36.40%	8.75%	17.63%	26.80%	36.40%
SS	86.48	71.19	56.46	51.40	71.30	54.15	39.10	35.80	52.89	37.17	28.50	24.31
LS	55.52	40.92	33.33	31.79	34.79	26.40	23.79	23.16	31.79	19.73	12.52	11.24
MS	37.77	28.17	20.57	16.14	15.72	14.30	10.18	7.26	26.16	16.19	11.57	9.57
US	25.01	19.54	16.60	12.62	9.30	8.63	8.00	6.15	17.32	11.94	9.34	6.90

The sediment yield and runoff volume also differed among different slope gradients. In contrast to CK, the total runoff volume with a slope gradient of 8.75% under the T1 treatment for SS, LS, MS, and US decreased by 84.67%, 53.95%, 35.10%, and 22.91%, respectively, and those under the T2 treatment for SS, LS, MS, and US decreased by 43.66%, 32.70%, 22.77%, and 16.58%, respectively. In contrast to CK, the sediment yield with a slope gradient of 8.75% under the T1 treatment for SS, LS, MS, and US decreased by 86.48%, 55.52%, 37.77%, and 25.01%, respectively, and those under the T2 treatment for SS, LS, MS, and US decreased by 71.30%, 34.79%, 15.72%, and 9.30%, respectively. In contrast to CK, the total runoff volume with a slope gradient of 17.63%, 26.80%, and 36.40% for the T1 treatment decreased by 16.85% to 67.35%, 11.06% to 52.86%, and 7.19% to 44.20%, respectively, and those for the T2 treatment decreased by 11.48% to 39.02%, 8.72% to 36.14%, and 6.16% to 34.01%, respectively. In contrast to CK, the sediment yield with slope gradients of 17.63%, 26.80%, and 36.40% for the T1 treatment decreased by 19.54% to 71.19%, 16.60% to 56.46%, and 12.62% to 51.40%, respectively, and those for the T2 treatment decreased by 8.63% to 54.15%, 8.00% to 39.10%, and 6.15% to 35.80%, respectively.

### 3.4 Contribution rates from vegetation component in RRB and SRB

**Figure 7:**
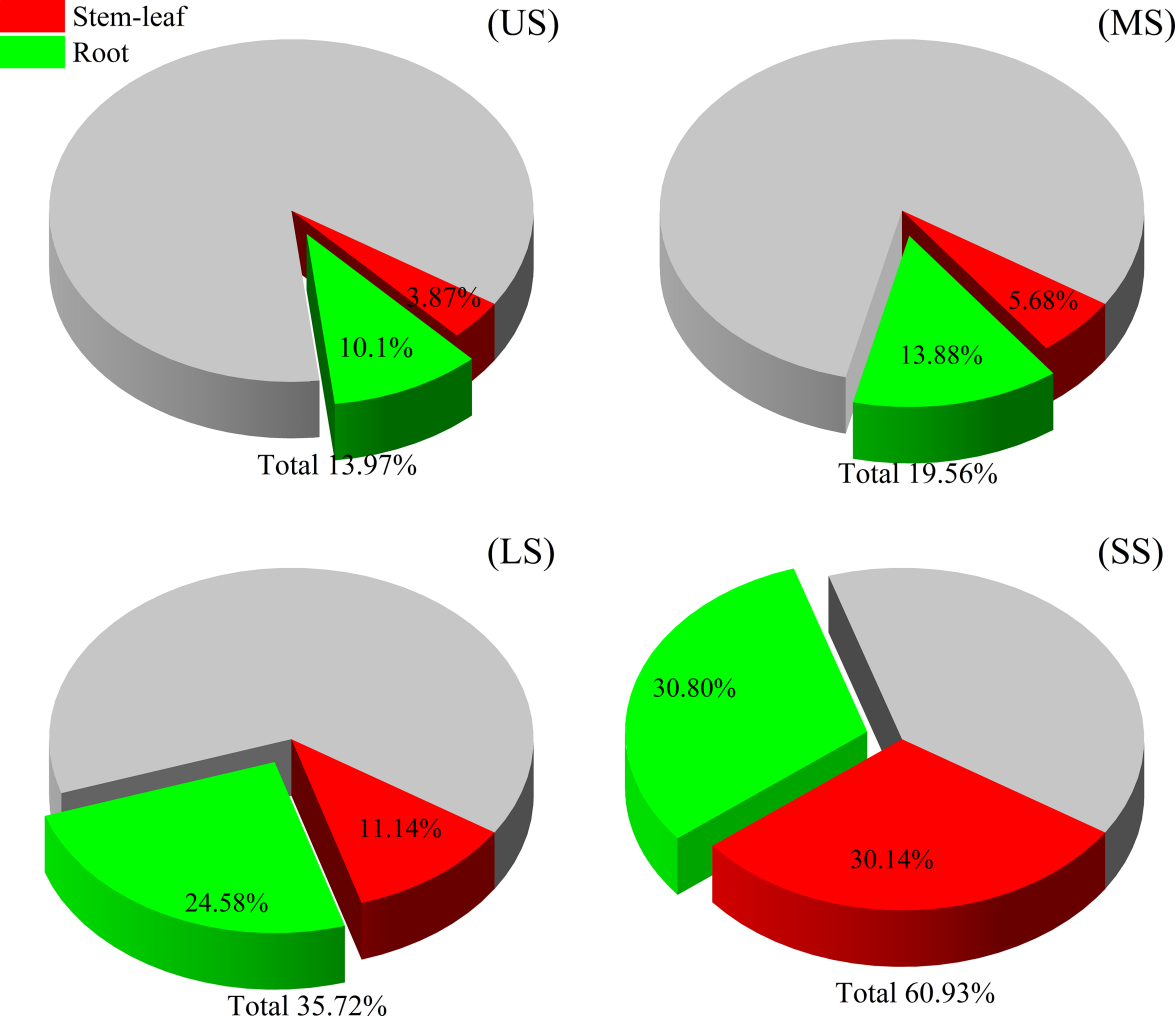
Contribution of stem–leaf and root of alfalfa to runoff reduction.

**Figure 8:**
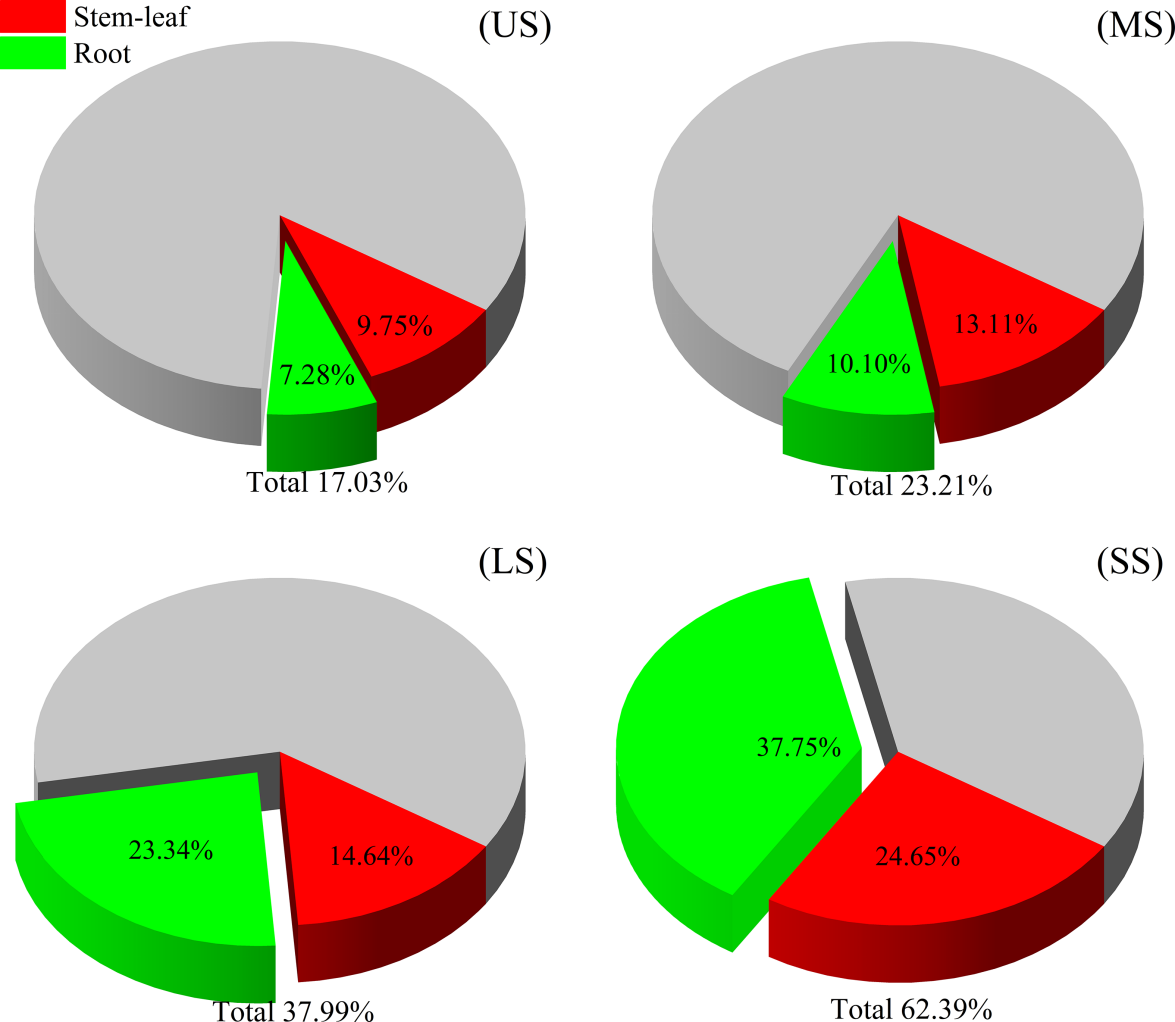
Contribution of stem–leaf and root of alfalfa to sediment reduction.

The RRB and SRB differed from the alfalfa vegetation pattern and vegetation components (**Figures 7**, **8**). The total contribution from the alfalfa grassland to RRB for SS, LS, MS, and US was 60.93%, 35.72%, 19.56%, and 13.97%, respectively, which from the stem–leaf ranged from 3.87% to 30.14% and that from the roots ranged from 10.10% to 30.80%. The total contribution of alfalfa grassland to SRB for SS, LS, MS, and US was 62.39%, 37.99%, 23.21%, and 17.03%, respectively, which from the stem–leaf ranged from 9.75% to 24.65% and that from the root ranged from 7.28% to 37.75%. The stem–leaf contribution to SRB was inconsistent with that of roots under different conditions. The root contribution to SRB for SS and LS was higher than that of the stem–leaf contribution but for MS and US was the opposite.

## 4 Discussion

### 4.1 Differences in soil erosion process for different vegetation spatial distribution patterns

In the current study, the soil erosion process varied with vegetation spatial distribution pattern. For alfalfa with the US pattern, the alfalfa was located at the upper part of the hillslopes, and the rainfall was shielded by alfalfa in the upper area of the hillslopes (Li et al., 2024a; Niu et al., 2024). On the contrary, in the absence of obstruction and shielding provided by vegetation, the runoff velocity and raindrop splash were higher in 50% of the area downstream of the hillslopes (Ma et al., 2015; Zhu et al., 2024b). Thus, the soil erosion rate maintained a higher level for the US pattern. For alfalfa with the MS pattern, the runoff and sediment rates were enhanced in the lower and upper 25% areas of the hillslopes without vegetation coverage (Feng et al., 2018; Li et al., 2024a; Zhang et al., 2014). The runoff and sediment produced in the upper 25% area of the hillslopes were obstructed by alfalfa in the middle of the hillslopes (Futerman et al., 2025; Yao et al., 2025; Zhang et al., 2024). Consequently, soil erosion with the MS pattern was lower than that with the US pattern. For alfalfa with the LS pattern, the runoff and sediment produced in the upper area of the hillslopes were retained by alfalfa located in the downstream area of the hillslopes (Futerman et al., 2025; Lin et al., 2025; Peng et al., 2026). Thus, soil erosion was reduced and maintained at a lower level. For the SS pattern, alfalfa was uniformly distributed on the hillslopes, and a multi-level protection system in soil erosion reduction has been established (Xia et al., 2025; Zhang et al., 2024). The runoff and sediment were retained by the uniformly distributed alfalfa (Dai et al., 2024; Oliveira et al., 2024; Yubonchit et al., 2025). The vegetation coverage of 50% on hillslopes with an effective soil erosion prevention function has been proven in a previous study, and the SS pattern showed the best efficiency (Yao et al., 2025; Zi et al., 2024). Vegetation was evenly distributed on the hillslopes, which could evenly intercept rain, block runoff, and stabilize the soil structure through the root systems across the entire hillslopes (Knapen et al., 2007; Li et al., 2024a). It comprehensively mitigated soil erosion by reducing raindrop splashing, slope runoff scouring, and enhancing soil stability (Shuai et al., 2025; Wang et al., 2022a). Therefore, the effect of erosion reduction and enhancing soil stabilization for the SS pattern was better. However, the vegetation located in the upper and lower hillslopes would leave large exposed areas, which were prone to cause concentrated flow erosion and heavy raindrop impacts, greatly reducing the anti-erosion effect of the vegetation (Liu et al., 2022; Van Dijk and Bruijnzeel, 2001; Wang et al., 2022b).

The soil erosion process also differed for CK and the T1 and T2 treatments, and the soil erosion rate presented a decreasing order in CK, T2, and T1. The difference in CK and the T1 and T2 treatments may be induced by vegetation coverage and the functional discrepancy of vegetation components (Dupuy et al., 2012; Feng et al., 2018; Li et al., 2024a). Since there was a lack of the shielding effect provided by vegetation and the soil-stabilizing function of roots for CK, the soil mass was directly impacted by raindrop, and runoff velocity was higher in the hillslopes, leading to higher runoff energy and severer concentrated flow erosion (Knapen et al., 2007, 2008; Tuo et al., 2025). Only the root system anchoring function was retained, and the shielding effect provided by vegetation was absent in the T2 treatment (Yao et al., 2023). On the contrary, the protection effect from vegetation coverage lowered the runoff velocity and enhanced the anti-erosion ability for T1 treatment (Wang et al., 2025b; Zhou et al., 2025). Furthermore, complete vegetation component, including root system and above-ground biomass for the T1 treatment, was beneficial for reducing soil erosion (Song et al., 2026; Zhu et al., 2026). In the current study, the contribution rate of 3.87% to 30.14%% from stem–leaf and the contribution rate of 10.10% to 37.75% from root to RRB and SRB also proved that complete vegetation component could reduce soil erosion effectively.

### 4.2 Factor influencing soil erosion

**Figure 9:**
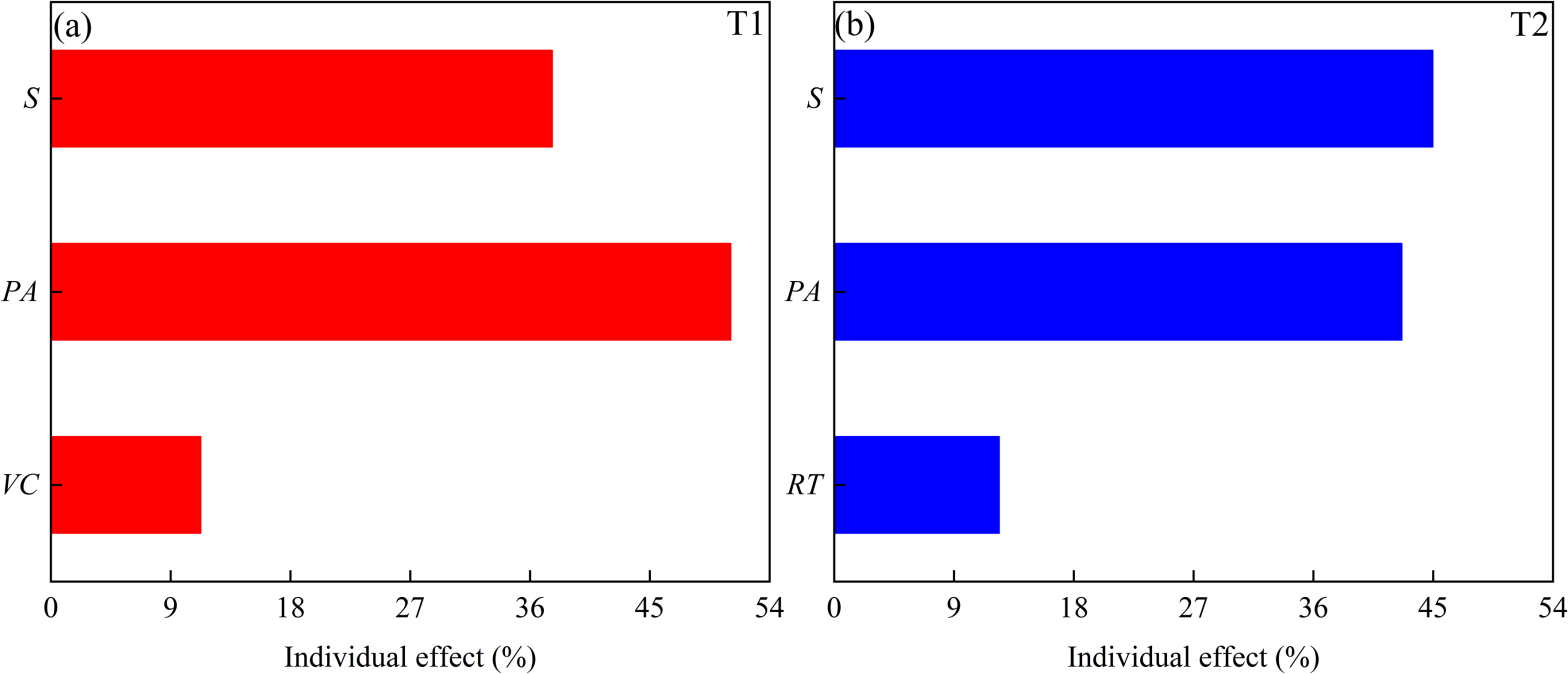
Individual effects of slope gradient (*S*), alfalfa spatial distribution pattern (PA), vegetation cover (VC), and root (RT) on runoff volume for T1 treatment **(a)** and T2 treatment (**b**).

The measured soil erosion process indexes, including runoff rate, sediment rate, infiltration rate, differed for CK and the T1 and T2 treatments, which may be induced by the difference in slope gradient, vegetation spatial distribution pattern, vegetation coverage, and root system (Chen et al., 2024; Feng et al., 2018; Hu et al., 2023). In this study, the runoff volume was selected to analyze the influence of individual factor, and VPA was conducted. The results presented showed that slope gradient, vegetation spatial distribution pattern, and vegetation coverage explained the variation in soil erosion of 36.67%, 51.08%, and 11.27% for the T1 treatment and that slope gradient, vegetation spatial distribution pattern, and root system explained the variation in soil erosion of 44.98%, 42.65%, and 12.40% for the T2 treatment (**Figure 9**). The difference in the T1 and T2 treatments might be induced by the absence of above-ground biomass (Hu et al., 2023; Yao et al., 2023).

Slope gradient played a key role in regulating soil erosion (Chen et al., 2024; Lin et al., 2019). In the current study, the contribution to soil erosion from slope gradient for CK and the T1 and T2 treatments accounted for more than 35%. The soil erosion rate was increased with slope gradient. The runoff energy linearly or exponentially increased with slope gradient (Parhizkar et al., 2025; Shen et al., 2021a, 2021). More severe concentrated flow erosion occurred with higher runoff energy, generating higher runoff and sediment rates (Bautista et al., 2007; Tuo et al., 2025). In addition, slope gradient demonstrated a core role in regulating splash erosion (Wang et al., 2024e). The splashed soil particles were evenly dispersed in all directions on flat ground, and soil erosion risk was relatively low (Wang et al., 2024b). As the slope gradient increased, the splashed soil particles along downstream hillslopes were more than those splashed along upper hillslopes, increasing the splashed soil particles moving downward of the hillslopes (Li et al., 2024b; Wang et al., 2025a). Therefore, soil erosion showed a significant increasing trend with slope gradient.

The different vegetation spatial distribution pattern demonstrated various functions on reducing runoff velocity and soil erosion prevention (Dai et al., 2024; Feng et al., 2018). In the current study, alfalfa with vegetation coverage of 50% were located in different positions for the US, MS, LS, and SS patterns, and the contribution from the vegetation spatial distribution patterns for CK and the T1 and T2 treatments accounted for more than 40%. The shielding effect of vegetation on rainfall impact and the reducing effect on runoff velocity from alfalfa coverage for the US and MS patterns were absent in 50% and 25% of the downstream hillslope areas, respectively. The alfalfa with vegetation coverage of 50% located at the downstream area of the hillslopes effectively reduced the overland flow erosion and minimized the sediment generated from the bare area on the upper area of the hillslopes (Song et al., 2026; Tuo et al., 2025). The alfalfa with vegetation coverage of 50% was evenly distributed on the hillslopes, and the ground vegetation coverage effectively reduced the impact of direct raindrops (Yao et al., 2025). The stems and leaves minimized the runoff velocity and energy (Niu et al., 2024; Wang et al., 2023). Meanwhile, the widely distributed root system enhanced the permeability of the hillslopes, and the root exudates improved the soil stability (Nardi et al., 2002; Niu et al., 2024). The vegetation coverage and roots regulated soil erosion by shielding effect, root fixation, and root exudates (Nardi et al., 2002; Saadati et al., 2023). In current study, the vegetation coverage and root system only explained the variation in soil erosion accounting for 11.27% and 12.40% for the T1 and T2 treatments, respectively. In terms of the results for the same vegetation coverage and root density in the US, MS, LS, and SS patterns, the variation in vegetation coverage and roots is relatively small. Thus, the vegetation coverage and root systems revealed less information compared with slope gradient and vegetation spatial distribution pattern.

### 4.3 Significance and limitation

This study investigated the influence of typical forage grass on the soil erosion process and quantified the contribution rate of alfalfa stem–leaf and root to soil erosion reduction benefits. The research results showed that the soil erosion process varied with the vegetation spatial distribution pattern, and the contribution rate of alfalfa stems–leaf and root to the sediment reduction benefits varied with different vegetation distribution patterns. For the runoff reduction benefits, the contribution rate of root systems was higher than that of stem–leaf. This study clarified the influence mechanism of vegetation spatial distribution and vegetation components on soil erosion, offering a reference for innovative policy formulation and soil erosion reduction. Although the study achieved good progress, the vegetation communities typically contain a variety of herbaceous plants, and the coverage vegetation and root systems also change with plants’ growth for the actual field environment. Only the impact of alfalfa on soil erosion during flowering period was investigated in the current study. The impact of composite vegetation types and vegetation succession on soil erosion still requires further investigation. Furthermore, the findings of this study should be validated in other soil-erosion-prone areas beyond the Loess Plateau.

## 5 Conclusion

The rainfall simulations were carried out on runoff plots with diverse alfalfa spatial distribution pattern. In contrast to CK, the initial runoff generation time for the T1 and T2 treatments was delayed. The mean runoff rate of the T1 and T2 treatments ranged from 0.18 to 1.15 and 0.61 to 1.16 L m^−2^ min^−1^, respectively. The mean sediment rate of the T1 and T2 treatments ranged from 1.50 to 18.63 and from 3.01 to 20.01 g m^−2^ min^−1^, respectively. In contrast to CK, the mean infiltration rate for the T1 and T2 treatments increased by 31.39% to 237.82% and by 21.39% to 188.33%, respectively. This indicates that the alfalfa spatial distribution pattern and the components have different regulatory effects on the soil erosion process. The contribution from alfalfa with diverse spatial distribution patterns to runoff reduction was 13.97% to 60.93%, and the contribution rate from stem–leaf ranged from 3.87% to 30.14% and that for roots ranged from 10.10% to 30.80%. The total contribution of alfalfa grassland with diverse spatial distribution patterns to the reduction in sediment was 17.03% to 62.39%, respectively, and the contribution rates from stem–leaf ranged from 9.75% to 24.65% and those from the roots ranged from 7.28% to 37.75%. Principal component analysis indicated that the slope gradient explains the greatest variation in runoff, followed by the spatial distribution pattern and vegetation traits. The findings provide valuable insights for soil erosion reduction in the Loess Plateau and selecting optimal soil and water conservation strategies. This information can be utilized for efficient water resource management and soil erosion control in arid and semi-arid regions.

## Data Availability

The original contributions presented in the study are included in the article/supplementary material. Further inquiries can be directed to the corresponding author.

## References

[B1] Bautista S. Mayor Á.G. Bourakhouadar J. Bellot J. (2007). Plant spatial pattern predicts hillslope runoff and erosion in a semiarid Mediterranean landscape. Ecosystems 10, 987–998. doi: 10.1007/s10021-007-9074-3. PMID: 30311153

[B2] Chen R. Dou H. Lin Y. Liu Q. Jian W. (2024). In-situ infiltration-runoff characterization of slopes under the influences of different rainfall patterns and slope gradients. Catena 247, 108519. doi: 10.1016/j.catena.2024.108519. PMID: 38826717

[B3] Dai W. Feng G. Huang Y. Tewolde H. Shankle M. W. Jenkins J. N. (2024). Soil aggregate stability and erosion resistance in response to integration of cover crops and poultry litter in a no-till rainfed soybean cropping system. Soil Tillage Res. 244, 106245. doi: 10.1016/j.still.2024.106245. PMID: 38826717

[B4] De Baets S. Poesen J. Knapen A. Galindo P. (2007). Impact of root architecture on the erosion‐reducing potential of roots during concentrated flow. Earth Surf. Processes Landforms 32, 1323–1345. doi: 10.1002/esp.1470. PMID: 41531421

[B5] Di Prima S. Fernandes G. Burguet M. Salazar M. P. Marras E. Murgia I. . (2026). Trees control hillslope subsurface flow: Insights from stemflow and throughfall experiments, geophysical surveys, and numerical modeling. J. Hydrol. 665, 134723. doi: 10.1016/j.jhydrol.2025.134723. PMID: 38826717

[B6] Dupuy J. M. Hernández-Stefanoni J. L. Hernández-Juárez R. A. Tetetla-Rangel E. López-Martínez J. O. Leyequién-Abarca E. . (2012). Patterns and correlates of tropical dry forest structure and composition in a highly replicated chronosequence in Yucatan, Mexico. Biotropica 44, 151–162. doi: 10.1111/j.1744-7429.2011.00783.x. PMID: 40046247

[B9] Feng X. M. Fu B. J. Piao S. Wang S. H. Ciais P. Zeng Z. Z. . (2016). Revegetation in China’s Loess Plateau is approaching sustainable water resource limits. Nat. Clim. Change 6, 1019–101+. doi: 10.1038/nclimate3092. PMID: 37880705

[B8] Feng T. Wei W. Chen L. Rodrigo-Comino J. Die C. Feng X. . (2018). Assessment of the impact of different vegetation patterns on soil erosion processes on semiarid loess slopes. Earth Surf. Processes Landforms 43, 1860–1870. doi: 10.1002/esp.4361. PMID: 41531421

[B7] Feng H. J. Jin J. M. (2024). Responses of vegetation to hydroclimatic variables on the Loess Plateau after large scale vegetation restoration. Hydrol. Processes 38, e15283. doi: 10.1002/hyp.15283. PMID: 41531421

[B10] Fu Y. Li G. Zheng T. Zhao Y. Yang M. (2020). Fragmentation of soil aggregates induced by secondary raindrop splash erosion. Catena 185, 104342. doi: 10.1016/j.catena.2019.104342. PMID: 38826717

[B11] Futerman S. I. Cohen Y. Laor Y. Argaman E. Aharon S. Eshel G. (2025). Assessing field-scale rill erosion mitigation by cover crops in arable land using drone image analysis. Soil Tillage Res. 246, 106341. doi: 10.1016/j.still.2024.106341. PMID: 38826717

[B12] Gyssels G. Poesen J. Bochet E. Li Y. (2005). Impact of plant roots on the resistance of soils to erosion by water: a review. Prog. Phys. Geogr. 29, 189–217. doi: 10.1016/b978-0-12-820106-0.00016-6. PMID: 38826717

[B13] Hu Y. Zhang F. Luo Z. Badreldin N. Benoy G. Xing Z. (2023). Soil and water conservation effects of different types of vegetation cover on runoff and erosion driven by climate and underlying surface conditions. Catena 231, 107347. doi: 10.1016/j.catena.2023.107347. PMID: 38826717

[B14] Huang W. Wang P. He L. Liu B. (2023). Improvement of water yield and net primary productivity ecosystem services in the Loess Plateau of China since the “Grain for Green” project. Ecol. Indic. 154, 110707. doi: 10.1016/j.ecolind.2023.110707. PMID: 38826717

[B15] Knapen A. Poesen J. Govers G. Baets S. D. (2008). The effect of conservation tillage on runoff erosivity and soil erodibility during concentrated flow. Hydrol. Processes 22, 1497–1508. doi: 10.1002/hyp.6702. PMID: 41531421

[B16] Knapen A. Poesen J. Govers G. Gyssels G. Nachtergaele J. (2007). Resistance of soils to concentrated flow erosion: a review. Earth Sci. Rev. 80, 75–109. doi: 10.1016/j.earscirev.2006.08.001. PMID: 38826717

[B17] Leung A. K. Boldrin D. Liang T. Wu Z. Y. Kamchoom V. Bengough A. G. (2017). Plant age effects on soil infiltration rate during early plant establishment. Geotechnique 68, 646–652. doi: 10.1680/jgeot.17.t.037

[B19] Li R. Fan B. Jin Z. Hao M. Liu H. Zhang Y. . (2024a). Divergent rainfall infiltration patterns on the Chinese Loess Plateau between growing and non-growing seasons after long-term revegetation. J. Hydrol. 641, 131816. doi: 10.1016/j.jhydrol.2024.131816. PMID: 38826717

[B20] Li X. Fan H. Xie F. Lei B. Ren G. (2024b). The role of soil dispersivity and initial moisture content in splash erosion: Findings from consecutive single-drop splash tests. Biosyst. Eng. 243, 27–41. doi: 10.1016/j.biosystemseng.2024.05.001. PMID: 38826717

[B18] Li Q. Liu G. B. Zhang Z. Tuo D. F. Bai R. R. Qiao F. F. (2017). Relative contribution of root physical enlacing and biochemistrical exudates to soil erosion resistance in the Loess soil. Catena 153, 61–65. doi: 10.1016/j.catena.2017.01.037. PMID: 38826717

[B21] Lin Q. T. Xu Q. Wu F. Q. Li T. T. (2019). Effects of wheat in regulating runoff and sediment on different slope gradients and under different rainfall intensities. Catena 183, 104196. doi: 10.1016/j.catena.2019.104196. PMID: 38826717

[B22] Lin X. Zhang S. Zhao X. Li R. Wang S. Yang L. . (2025). Global thresholds for the climate-driven effects of vegetation restoration on runoff and soil erosion. J. Hydrol. 647, 132374. doi: 10.1016/j.jhydrol.2024.132374. PMID: 38826717

[B23] Liu Y. Zhao L. Liu Y. Huang Z. Shi J. Wang Y. . (2022). Restoration of a hillslope grassland with an ecological grass species (Elymus tangutorum) favors rainfall interception and water infiltration and reduces soil loss on the Qinghai-Tibetan Plateau. Catena 219, 106632. doi: 10.1016/j.catena.2022.106632. PMID: 38826717

[B24] Ma B. Liu Y. X. Liu X. J. Ma F. Wu F. Q. Li Z. B. (2015). Soil splash detachment and its spatial distribution under corn and soybean cover. Catena 127, 142–151. doi: 10.1016/j.catena.2014.11.009. PMID: 38826717

[B25] Nardi S. Sessi E. Pizzeghello D. Sturaro A. Rella R. Parvoli G. (2002). Biological activity of soil organic matter mobilized by root exudates. Chemosphere 46, 1075–1081. doi: 10.1016/s0045-6535(01)00160-6. PMID: 11999770

[B26] Niu F. Pan C. Ma L. Cui Y. (2024). Efficacies of vegetation litter and roots in strengthening rainfall infiltration for different stand ages on the Loess Plateau. Catena 247, 108502. doi: 10.1016/j.catena.2024.108502. PMID: 38826717

[B27] Oliveira E. M. D. Hermógenes G. M. Brito L. D. C. Silva B. M. Avanzi J. C. Beniaich A. . (2024). Cover crop management systems improves soil quality and mitigate water erosion in tropical olive orchards. Sci. Hortic. 330, 113092. doi: 10.1016/j.scienta.2024.113092. PMID: 38826717

[B500] OriginLab Corporation (2021). OriginPro 2021. (Northampton, MA, USA: OriginLab Corporation).

[B28] Parhizkar M. Lucas-Borja M. E. Denisi P. Zema D. A. (2025) Effects of repeated low-severity fires on particle detachment capacity and soil properties in rills of semi-arid forests. Land Degrad. Dev. 36, 3698–3714. doi: 10.1002/ldr.5593

[B29] Peng D. Q. Du C. H. Xu R. L. Liu J. L. Zhang X. Y. Guo M. M. . (2026). Sustainable soil management: Rhizosphere microbial contributions to erosion control in herbaceous vegetation systems. Soil Tillage Res. 256, 106084. doi: 10.1016/j.still.2025.106844. PMID: 38826717

[B30] Puigdefábregas J. (2005). The role of vegetation patterns in structuring runoff and sediment fluxes in drylands. Earth Surf. Processes Landforms 30 (2), 133–147. doi: 10.1002/esp.1181

[B501] R Core Team (2021). R: A language and environment for statistical computing. (Vienna, Austria: R Foundation for Statistical Computing). Available online at: https://www.R-project.org/.

[B31] Saadati N. Mosaddeghi M. R. Sabzalian M. R. Jafari M. (2023). Soil mechanical reinforcement by the fibrous roots of selected rangeland plants using a large soil-root shear apparatus. Soil Tillage Res. 234, 105852. doi: 10.1016/j.still.2023.105852. PMID: 38826717

[B32] Shen E. Liu G. Dan C. Shu C. Wang R. Liu X. . (2021a). Combined effects of rainfall and flow depth on the resistance characteristics of sheet flow on gentle slopes. J. Hydrol. 603, 127112. doi: 10.1016/j.jhydrol.2021.127112. PMID: 38826717

[B33] Shen N. Wang Z. L. Guo Q. Zhang Q. W. Wu B. Liu J. . (2021b). Soil detachment capacity by rill flow for five typical loess soils on the Loess Plateau of China. Soil Tillage Res. 213, 105159. doi: 10.1016/j.still.2021.105159. PMID: 38826717

[B34] Shuai F. Wu W. Meng Y. Zhou Y. Y. Chen Y. Y. Zhang Y. . (2025). Effect of shrub root diameter classes on shear strength of soil in Benggang collapsing walls. Earth Surf. Processes Landforms 50, e70128. doi: 10.1002/esp.70128. PMID: 41531421

[B35] Song Y. Yao Y. F. Kong W. B. Guo L. C. Bao K. Q. Qiu L. P. . (2026). Effects of vegetation loss and soil erosion intensity on soil carbon dynamics across landscape position: Evidence from China’s Loess Plateau. Agric. Ecosyst. Environ. 396, 109992. doi: 10.1016/j.agee.2025.109992. PMID: 38826717

[B36] Tuo M. Qiao H. L. Xu G. C. Wang B. Wan S. Wang X. N. . (2025). Effects of vegetation types on hillslope runoff and soil erosion on the Loess Plateau. Catena 260, 109487. doi: 10.1016/j.catena.2025.109487. PMID: 38826717

[B37] Van Dijk A. I. J. M. Bruijnzeel L. A. (2001). Modelling rainfall interception by vegetation of variable density using an adapted analytical model. Part 2. Model validation for a tropical upland mixed cropping system. J. Hydrol. 247, 239–262. doi: 10.1016/s0022-1694(01)00393-6

[B50] Wang X. A. Chen X. Chen H. Zhao W. (2024d). Influence mechanism of herbaceous plants on debris flow bank erosion. Catena 245, 108308. doi: 10.1016/j.catena.2024.108308. PMID: 38826717

[B40] Wang C. Li Z. Cai B. Tan Q. Li Y. He L. . (2022a). Effect of root system of the Dicranopteris dichotoma on the soil unconfined compressive strength of collapsing walls in hilly granite area of South China. Catena 216, 106411. doi: 10.1016/j.catena.2022.106411. PMID: 38826717

[B42] Wang C. F. Li F. C. Wang J. Zhang X. M. Wang X. P. Wang Y. Q. . (2025a). Understanding the interaction between raindrop splash and concentrated flow on soil detachment capacity across different slope gradients. Catena 258, 109252. doi: 10.1016/j.catena.2025.109252. PMID: 38826717

[B43] Wang D. Liu C. Yang Y. Liu P. Hu W. Song H. . (2023). Clipping decreases plant cover, litter mass, and water infiltration rate in soil across six plant community sites in a semiarid grassland. Sci. Total Environ. 861, 160692. doi: 10.1016/j.scitotenv.2022.160692. PMID: 36476773

[B44] Wang J. F. Liu G. B. Yang Y. F. Wang B. (2026). Construction of a comprehensive root parameter to reflect the effect of plant root systems on soil erosion. Land Degrad. Dev. doi: 10.1002/ldr.70456. PMID: 41531421

[B41] Wang C. Ma J. Wang Y. Li Z. Ma B. (2022b). The influence of wheat straw mulching and straw length on infiltration, runoff and soil loss. Hydrol. Processes 36, e14561. doi: 10.1002/hyp.14561. PMID: 41531421

[B49] Wang R. Qin C. Sun H. Feng Y. (2024c). Effects of root morphologies on shearing characteristics of the root-soil composite: An experimental case study of Ficus virens in Chongqing, China. Catena 246, 108407. doi: 10.1016/j.catena.2024.108407. PMID: 38826717

[B45] Wang J. F. Yang Y. F. Wang B. Liu G. B. (2024a). The role of near-surface vegetation in modulating overland flow resistance in grasslands of the Chinese Loess Plateau. Earth Surf. Processes Landforms 49, 3541–3554. doi: 10.1002/esp.5921. PMID: 41531421

[B46] Wang J. F. Yang Y. F. Wang B. Liu G. B. (2025b). Effects of vegetation restoration on rill erosion on the Chinese Loess Plateau. Land Degrad. Dev. 36, 4359–4371. doi: 10.1002/ldr.5639. PMID: 41531421

[B47] Wang J. F. Yang Y. F. Wang B. Liu G. B. (2025c). Seasonal variations in soil erosion resistance under tap and fibrous root systems grasslands on the Chinese Loess Plateau. Geoderma 458, 117350. doi: 10.1016/j.geoderma.2025.117350. PMID: 38826717

[B38] Wang B. Zhang G. H. (2017). Quantifying the binding and bonding effects of plant roots on soil detachment by overland flow in 10 typical grasslands on the Loess Plateau. Soil Sci. Soc Am. J. 81, 1567–1576. doi: 10.2136/sssaj2017.07.0249

[B48] Wang L. Zhang C. Peng J. Xu L. Wang J. Cai C. (2024b). Splash erosion-induced soil aggregate turnover and associated organic carbon dynamics. Soil Tillage Res. 235, 105900. doi: 10.1016/j.still.2023.105900. PMID: 38826717

[B39] Wang B. Zhang G. H. Shi Y. Y. Zhang X. C. (2014). Soil detachment by overland flow under different vegetation restoration models in the Loess Plateau of China. Catena 116, 51–59. doi: 10.1016/j.catena.2013.12.010. PMID: 38826717

[B51] Wang Z. Zhang Q. Zhang Z. Lu C. Wu F. (2024e). Effects of tillage microrelief units on splash erosion: A case study from the Loess Plateau, in China. Soil Tillage Res. 238, 106004. doi: 10.1016/j.still.2024.106004. PMID: 38826717

[B52] Xia Y. Y. Li W. Y. Jiang L. S. Gan F. L. Yan Y. J. Fan Y. C. . (2025). Assessing soil detachment driven by root-soil interactions in karst trough valleys: Influence of vegetation restoration on erosion-deposition contrasts. Research Square. doi: 10.21203/rs.3.rs-7451615/v1. PMID: 42160304

[B53] Xiao Y. Zhang J. Jia T. T. Pang X. P. Guo Z. G. (2015). Effects of alternate furrow irrigation on the biomass and quality of alfalfa (Medicago sativa). Agric. Water Manage. 161, 147–154. doi: 10.1016/j.agwat.2015.07.018. PMID: 38826717

[B54] Yang J. Ma H. Zhang R. Ji W. (2025). Effects of “Grain for Green” program on soil hydraulic properties: A meta-analysis. Geoderma 453, 117130. doi: 10.1016/j.geoderma.2024.117130. PMID: 38826717

[B55] Yao C. Chen K. Zhang Q. (2023). The contribution rate of stem-leaf and root of alfalfa (Medicago sativa L.) to sediment and runoff reduction. Land Degrad. Dev. 34 (13). doi: 10.1002/ldr.4731. PMID: 41531421

[B57] Yao C. Zhang Q. W. Chen K. B. Zhang S. G. Zhu M. Gu Z. J. . (2025). Quantifying the impacts of diverse vegetation-covered patterns on hillslope soil erosion: A case experiment of alfalfa-covered hillslopes. Front. Plant Sci. 16. doi: 10.3389/fpls.2025.1629542. PMID: 40926797 PMC12415054

[B56] Yao C. Zhang Q. Zhuan Y. Fu S. Lin H. Wang H. . (2026). Rainfall-induced soil physical crust formation reduces soil detachment capacity by enhancing soil erosion resistance. Land Degrad. Dev. doi: 10.1002/ldr.70433. PMID: 41531421

[B58] Yibin W. Fei L. Jian W. Hongyu C. Mengfei L. (2024). The social-ecological benefits of grain for green program based on coupled coordination network: Taking the China’s Loess Plateau as an example. Land Use Policy 143, 107211. doi: 10.1016/j.landusepol.2024.107211. PMID: 38826717

[B59] Yubonchit S. Chinkulkijniwat A. Tirametatiparat T. Horpibulsuk S. (2025). Rainwater infiltration in a vegetated slope subjected to high intensity rainfall. Phys. Chem. Earth Parts A/B/C 138, 103841. doi: 10.1016/j.pce.2024.103841. PMID: 38826717

[B62] Zhang G. Liu G. Zhang P. Yi L. (2014). Influence of vegetation parameters on runoff and sediment characteristics in patterned Artemisia capillaris plots. J. Arid. Land 6, 352–360. doi: 10.1007/s40333-013-0224-5. PMID: 30311153

[B63] Zhang P. Xing G. Hu X. Liu C. Li X. Zhao J. . (2024). Effects of grassland vegetation roots on soil infiltration rate in Xiazangtan super large scale landslide distribution area in the upper reaches of the Yellow River, China. Biogeotechnics 2, 100104. doi: 10.1016/j.bgtech.2024.100104. PMID: 38826717

[B60] Zhang B. J. Zhang G. H. Yang H. Y. Zhu P. Z. (2019a). Temporal variation in soil erosion resistance of steep slopes restored with different vegetation communities on the Chinese Loess Plateau. Catena 182, 104170. doi: 10.1016/j.catena.2019.104170. PMID: 38826717

[B61] Zhang B. J. Zhang G. H. Zhu P. Z. Yang H. Y. (2019b). Temporal variations in soil erodibility indicators of vegetation-restored steep gully slopes on the Loess Plateau of China. Agric. Ecosyst. Environ. 286, 106661. doi: 10.1016/j.agee.2019.106661. PMID: 38826717

[B64] Zhou Z. C. Zheng Q. W. Chen M. Y. Wang N. Liu J. Zhu B. B. (2025). Response of soil detachment and erodibility to perennial fibrous-rooted vegetation coverage (Stipa bungeana) on the Loess Plateau. Catena 255, 109043. doi: 10.1016/j.catena.2025.109043. PMID: 38826717

[B66] Zhu Z. Li J. Zhu D. (2024a). Exploring the effects of maize canopy on the spatiotemporal distribution heterogeneity of the determinants of sprinkler irrigation droplet splash erosivity. Agric. Water Manage. 306, 109158. doi: 10.1016/j.agwat.2024.109158. PMID: 38826717

[B67] Zhu Z. Li J. Zhu D. Gao Z. (2024b). The impact of maize canopy on splash erosion risk on soils with different textures under sprinkler irrigation. Catena 234, 107608. doi: 10.1016/j.catena.2023.107608. PMID: 38826717

[B65] Zhu R. P. Zhang G. H. Xing S. K. (2026). Soil-root shear strength of gullies covered by different vegetation types on the Loess Plateau of China. Land Degrad. Dev. doi: 10.1002/ldr.70435. PMID: 41531421

[B68] Zi R. Zhao L. Fang Q. Fang F. Yin X. Qian X. . (2024). Effect of Nostoc commune cover on shallow soil moisture, runoff and erosion in the subtropics. Geoderma 447, 116931. doi: 10.1016/j.geoderma.2024.116931. PMID: 38826717

